# Exploring Free Will Beliefs and Attitudes Towards Punishment in Norway, Sweden and the United Kingdom

**DOI:** 10.3390/bs16060868

**Published:** 2026-05-30

**Authors:** Haakon Solum, Zoe Stephenson, Stephanie Wilson, Alexander Jack

**Affiliations:** 1 School of Psychology, Edgbaston Campus, University of Birmingham, Birmingham B15 2TT, UK; hss067@alumni.bham.ac.uk (H.S.); s.wilson.9@bham.ac.uk (S.W.); 2 Department of Psychology, University of Bath, Bath BA2 7AY, UK; aj2474@bath.ac.uk

**Keywords:** free will, moral responsibility, punitive attitudes, sentencing preferences, criminal justice systems, cross-cultural comparison

## Abstract

Beliefs about free will shape how individuals judge moral responsibility and support different forms of punishment. Most research has relied on single-country samples, limiting understanding of how cultural and institutional contexts might influence these beliefs. Therefore, this study compared free will beliefs and punitive attitudes across three nations with contrasting justice traditions: Norway and Sweden, which emphasise rehabilitation and are more compatible with determinist perspectives, and the United Kingdom, which has historically incorporated stronger libertarian and retributive elements. A total of 328 participants (98 Norwegian, 115 Swedish, 115 British) completed measures of libertarian free will, determinism, and dualism, alongside a task assessing preferred severity of punishment for a hypothetical crime. The results showed that British participants scored higher on libertarian free will and dualism, whereas Norwegian and Swedish participants reported lower overall free will beliefs and selected less severe punishments. Across the full sample, stronger belief in free will was associated with harsher sentencing preferences, while associations involving determinism and dualism were more varied. These findings indicate that national context may play a role in shaping free will beliefs and punishment attitudes, suggesting that both cultural and institutional features could contribute to differences in how individuals judge responsibility.

## 1. Introduction

Most people endorse some form of free will belief (Nadelhoffer et al., 2014; Nahmias et al., 2005), and these beliefs appear to shape how responsibility, blame, and punishment are understood (Martin et al., 2017; Monroe et al., 2016). Stronger belief in free will has been associated with greater support for punitive responses, whereas weaker belief has been associated with less punitive attitudes (Appelbaum et al., 2015; Stinnett & Alquist, 2022). This relationship is important because criminal justice systems depend, either explicitly or implicitly, on assumptions about agency and responsibility.

Debates about free will have long occupied philosophy and, more recently, psychology and neuroscience. Determinist and free will sceptic positions argue that behaviour is shaped by prior causes, including biology, environment, and learning history, such that the experience of freely choosing may be partly or wholly illusory (Bargh, 2008; Harris, 2012; Juth & Lorentzon, 2010; Sapolsky, 2023). By contrast, many legal systems are based on the assumption that individuals are sufficiently autonomous to be held responsible for their actions (Greene & Cohen, 2004). This assumption is especially relevant in criminal justice, where punishment is often justified on the basis that offenders could have acted otherwise.

In the present study, free will beliefs are understood in line with the Free Will Inventory (FWI; Nadelhoffer et al., 2014), which distinguishes between libertarian free will, determinism, and dualism. Libertarian free will refers to the belief that people can make genuinely free choices and could have acted otherwise in a given situation. Determinism refers to the belief that human behaviour is caused by prior events and conditions, such as biological, psychological, social, or environmental factors. Dualism refers to the belief that the mind, self, or soul is distinct from the physical body, meaning that human agency may not be fully reducible to physical or causal processes. These constructs are conceptually distinct but related, and previous research suggests that dualism may be particularly relevant to free will beliefs because it can preserve a sense of agency even when people recognise physical or environmental influences on behaviour (Nadelhoffer et al., 2014; Wisniewski et al., 2019).

The present study examines whether free will beliefs are associated with punitive attitudes and whether these beliefs vary across national contexts with differing criminal justice philosophies. Existing work has largely been conducted in countries with relatively retributive systems and strong assumptions of individual responsibility (Genschow et al., 2021). Less is known about whether similar relationships are observed in countries whose legal cultures place greater emphasis on rehabilitation, social causation, or consequentialist approaches to punishment.

Norway, Sweden, and the United Kingdom provide a useful comparison. These countries differ in how criminal responsibility and punishment are conceptualised, while remaining similar enough in broader social and democratic structures to make comparison meaningful. It is plausible that the wider institutional environment, including the criminal justice system, may help shape public beliefs over time. Institutional theorists have argued that social institutions influence norms, expectations, and beliefs (Jepperson & Meyer, 2021; Šubrt, 2022), and some free will research suggests that broader social environments may influence how responsibility and punishment are understood (Martin et al., 2017). Cross-national evidence also indicates that beliefs about free will can vary between countries with different legal and cultural contexts (Wisniewski et al., 2019).

The present study does not assume a simple one-way relationship in which criminal justice systems determine public beliefs. The relationship is more likely to be reciprocal and historically shaped. Nevertheless, if justice systems rest on particular assumptions about agency and moral responsibility, it is reasonable to ask whether those assumptions align with the beliefs of the populations they serve.

### 1.1. Moral Responsibility and Punishment

Criminal punishment is closely tied to ideas about moral responsibility. If people are seen as genuinely free and morally accountable, punishment may be justified as deserved. If behaviour is understood as heavily determined by social, psychological, or biological factors, then punishment may be justified less as retribution and more in terms of prevention, public protection, treatment, or rehabilitation.

Two broad approaches to punishment are especially relevant: retributivism and consequentialism (Fondacaro & O’Toole, 2015). Retributive punishment is grounded in the idea that offenders deserve punishment in proportion to their wrongdoing (Caruso, 2021). On this view, punishment is justified primarily by blameworthiness rather than by its future benefits. This position fits comfortably with stronger beliefs in free will and moral responsibility, because punishment is treated as a deserved response to a freely chosen wrong. It is important, however, not to equate retributivism with punishment severity. Retributive punishment may be severe, but in principle it is constrained by proportionality: punishment is justified by desert and should be proportionate to the wrongdoing (von Hirsch, 1992). Conversely, consequentialist reasoning can also support severe restrictions, for example where incapacitation is viewed as necessary to prevent future harm (Fondacaro & O’Toole, 2015). Thus, the distinction between retributive and consequentialist punishment concerns the justification for punishment, rather than severity alone.

In contrast, consequentialist punishment is justified by its outcomes rather than its moral symbolism. Its aims may include deterrence, incapacitation, rehabilitation, and the reduction in future harm. Rather than focusing on desert, consequentialist thinking places greater weight on whether an intervention reduces reoffending or protects society (Fondacaro & O’Toole, 2015; Greene & Cohen, 2004). This approach is more compatible with deterministic accounts of behaviour, in which offending is seen as arising from multiple interacting causes rather than from unconstrained choice alone.

These perspectives are not merely abstract philosophical positions; they have practical implications for sentencing, prison conditions, rehabilitation, and public attitudes. They may also shape how readily people accept harsh punishment. Koppel et al. (2018), for example, found that people who doubt free will tend to be more supportive of consequentialist punishment than those holding stronger libertarian views. Relatedly, Clark et al. (2017) argued that belief in free will may help alleviate “punitive distress” by making harsh punishment feel more morally defensible. If so, stronger free will beliefs may be especially useful in contexts where punishment is severe or explicitly retributive.

### 1.2. Criminal Justice Philosophy in the United Kingdom, Norway, and Sweden

Although all three countries punish criminal offending, they differ in the philosophical assumptions and penal practices that characterise their systems. The characterisations below are intended as broad, historically grounded comparisons rather than claims about fixed national character or current political attitudes alone. They are based on a combination of legal doctrine, stated sentencing aims, penal philosophy, prison practice, and comparative indicators such as sentence structure, prison conditions, and recidivism, while recognising that all three systems contain both retributive and consequentialist elements.

#### 1.2.1. The United Kingdom

The UK criminal justice system is strongly grounded in assumptions of individual responsibility. Legal doctrine generally treats competent adults as autonomous agents capable of choosing how to act. Sentencing in England and Wales explicitly includes punishment among its aims alongside crime reduction, rehabilitation, public protection, and reparation. This reflects a system in which punishment is not merely instrumental but also morally justified in its own right.

In practice, the UK system has often been characterised as comparatively punitive. Political rhetoric around being “tough on crime” has remained prominent, and despite stated commitments to rehabilitation, prisons in England and Wales have been criticised for overcrowding, low staffing, unrest, and limited rehabilitative success (Hale, 2020). Recidivism rates also remain relatively high (Yukhnenko et al., 2020). The comparative punitiveness of the UK context is also reflected in sentence structure: England and Wales allow life imprisonment, including whole-life orders, whereas Norway does not use life sentences and has a maximum determinate sentence of 21 years, although extensions are possible in exceptional cases (Benko, 2018; Howard League for Penal Reform, 2024; Prison Reform Trust, 2023). Taken together, the UK context appears broadly consistent with a system in which punishment retains a substantial retributive function, even if consequentialist aims are also present.

#### 1.2.2. Norway

Norwegian criminal law historically assumed that individuals are responsible for their actions, but the broader penal culture has increasingly incorporated more mechanistic and socially informed understandings of behaviour. While moral responsibility remains relevant, Norwegian penal practice is strongly oriented toward rehabilitation, reintegration, and harm reduction.

Norway is often cited as an example of a humane and rehabilitative penal model. The principle of normality holds that life in prison should resemble ordinary life as far as possible, with the loss of liberty being the punishment rather than the addition of unnecessary hardship. Restorative justice ideals have also been influential, with emphasis placed on repair, accountability, and reintegration rather than suffering for its own sake (Liebmann, 2007; Zehr et al., 1997). Norwegian officials have explicitly framed punishment as valuable only insofar as it leads to change and reduces future offending. Although international comparisons of recidivism must be treated cautiously, Norway is often reported as having comparatively low reconviction rates (Yukhnenko et al., 2020). At the same time, this lower recidivism should not be treated as straightforward evidence that the justice system alone is responsible. Norway’s wider social welfare context and generally low crime rates may also be relevant (Nilsen & Skarpenes, 2020; Statistics Norway, 2025), while international differences in reporting practices and definitions of recidivism complicate direct comparison (Fazel & Wolf, 2015).

This creates an interesting tension: Norway retains assumptions of responsibility, yet its penal practices are often more consequentialist and restorative than retributive.

#### 1.2.3. Sweden

Sweden differs more clearly from the UK in its philosophical starting point. The Swedish tradition has been strongly shaped by positivist criminology, with criminal behaviour often understood in more deterministic terms. Historically, the mental state of the offender has been treated less as a condition for criminal responsibility than as relevant to how the state should respond. In this model, crime is addressed less as a morally blameworthy choice and more as the product of individual and environmental factors requiring management, treatment, or prevention (Juth & Lorentzon, 2010; Levander, 2004).

In practice, the Swedish system has also been associated with the principle of normality, humane treatment, and imprisonment as a last resort. It has often been described as comparatively liberal, although there is evidence that confidence in this model has weakened and that Swedish penal policy has, at times, moved in a more punitive direction relative to its own history (Lappi-Seppälä, 2019; Ugelvik & Dullum, 2012). This rehabilitative orientation is also reflected in accounts of Swedish prison practice, including emphasis on normalisation, access to ordinary public services, and the use of imprisonment as a last resort (Lindström & Leijonram, 2008; Pettit & Kroth, 2011; Ugelvik & Dullum, 2012). Comparative prison indicators also suggest a less punitive institutional context in Norway and Sweden than in England and Wales, including lower prison population rates and lower inmate-to-staff ratios, although such indicators should be interpreted cautiously because definitions and data collection practices vary across countries (Aebi & Cocco, 2025). Still, the system remains more explicitly consequentialist than retributive in orientation, with a stronger focus on rehabilitation and social response than on desert alone.

### 1.3. Why These Differences May Matter

If criminal justice systems differ in how they conceptualise responsibility and punishment, public beliefs may also differ across these contexts. In more retributive systems, stronger free will beliefs may be especially useful in legitimising punishment, particularly where punishment involves substantial suffering or deprivation. In more consequentialist systems, by contrast, there may be less pressure to endorse strong free will beliefs, because punishment is justified less by desert and more by prevention, treatment, or social protection.

This does not mean that citizens simply absorb the philosophy of their justice system. Public beliefs may shape institutions just as institutions shape public beliefs, and both are influenced by wider cultural, political, and historical forces. Nor do these national comparisons imply that any of the three systems is philosophically pure or internally consistent; each combines elements of punishment, rehabilitation, public protection, and crime reduction. Nevertheless, examining whether public beliefs align with penal philosophy is theoretically valuable. It helps clarify whether criminal justice systems reflect broadly shared assumptions about human agency or operate on principles that may sit uneasily with public intuitions.

The comparison is also relevant because cross-national studies of free will and punishment remain limited. Much existing work has examined relationships between free will beliefs and punitive attitudes without considering whether those relationships vary across legal cultures. By comparing the UK, Norway, and Sweden, the present study explores whether belief in free will and support for punishment are embedded within broader institutional contexts rather than being psychologically uniform across settings.

### 1.4. Aims and Rationale

The present study investigates the relationship between free will beliefs and attitudes toward punishment across Norway, Sweden, and the United Kingdom. First, it examines whether stronger endorsement of free will is associated with harsher sentencing preferences and lower support for rehabilitative responses. Second, it examines whether participants from countries with differing criminal justice philosophies differ in their free will beliefs and punishment attitudes.

The study is guided by two broader questions. The first is whether free will beliefs predict punitive attitudes at the individual level. The second is whether these beliefs and attitudes vary systematically across national contexts whose justice systems differ in their assumptions about responsibility, punishment, and rehabilitation.

This is not a study of causality. It does not seek to show that criminal justice systems directly produce public beliefs, nor that public beliefs alone shape legal institutions. Rather, it examines whether there is alignment between individual beliefs and the broader philosophical orientation of the justice systems in which people live. Such alignment matters because assumptions about free will and responsibility help underpin penal policy. If those assumptions are weakly shared, this may have implications for how punishment is justified publicly and politically.

A better understanding of these relationships may also have practical relevance. If stronger belief in free will is associated with more punitive responses, and weaker belief with greater openness to rehabilitation, then beliefs about agency may play an underappreciated role in public support for different penal approaches. Understanding this may help explain resistance to, or support for, more humane and rehabilitative justice policies.

### 1.5. Choice of Countries

Norway, Sweden, and the United Kingdom were selected because they offer theoretically informative contrasts. The UK represents a system in which assumptions of individual responsibility and punitive sentencing remain relatively prominent. Norway represents a system that retains assumptions of responsibility while applying punishment in a more rehabilitative and restorative way. Sweden represents a context in which the justice system has been more explicitly shaped by deterministic and consequentialist ideas. Comparing these countries therefore makes it possible to examine whether public beliefs more closely reflect legal philosophy, penal practice, or some combination of the two.

At the same time, the study is limited in scope. These three countries do not capture the full range of global approaches to punishment, responsibility, or religious and cultural understandings of determinism. Some societies may combine harsh punishment with worldviews that appear more fatalistic or theologically deterministic. Exploring such contexts would be valuable, but was beyond the practical scope of the present study given issues of access, language, and comparability.

### 1.6. Hypotheses

1. **Nationality and free will beliefs**
  There will be significant differences between national groups in beliefs in libertarian free will, determinism, and dualism, as measured by the Free Will Inventory. Norwegian and Swedish participants are expected to report lower free will and dualism beliefs, and higher determinism, than British participants.

2. **Nationality as a predictor of Free Will Inventory scores after controlling for covariates**
  Participants’ scores on the Free Will Inventory subscales will be predicted by nationality, after controlling for demographic and personal-experience variables, with Norwegian and Swedish participants expected to score lower on free will and dualism and higher on determinism than British participants.

3. **Nationality and sentencing recommendations**
  British participants will be more likely than Norwegian and Swedish participants to recommend stricter punishments.

4. **Nationality and rehabilitation-related attitudes**
  Norwegian and Swedish participants will place greater importance than British participants on understanding why a person committed a crime and on access to treatment. They will also recommend less punitive sentences following rehabilitation.

5. **Free will beliefs and sentencing severity**
  Stronger free will beliefs will predict more severe sentencing recommendations.

6. **Free will beliefs and views on rehabilitation**
  Stronger free will beliefs will predict lower endorsement of understanding the causes of offending and lower endorsement of treatment for offenders.

7. **Free will beliefs and sentencing after rehabilitation**
  Stronger free will beliefs will predict harsher sentencing recommendations even after a hypothetical scenario in which an offender has completed a fully effective rehabilitation programme.

## 2. Materials and Methods

### 2.1. Design

The study used a cross-sectional, correlational design to examine associations between free will beliefs and attitudes towards punishment across three national groups: Norway, Sweden, and the United Kingdom.

### 2.2. Recruitment and Participants

Participants were recruited through online advertisements hosted on Prolific and shared via Facebook and Twitter/X, with most participants recruited through Prolific. Prolific recruitment was set up so that the study was advertised only to individuals who identified as Norwegian, Swedish, or British nationals. Participants were also screened on the first page of the survey to confirm that they met the eligibility criteria; those who did not identify as one of the three target nationalities were not able to proceed. Participants recruited through Prolific were compensated at a rate consistent with the UK minimum hourly wage at the time of data collection (£10.42 per hour; Low Pay Commission, 2023). Recruitment through Facebook and Twitter/X relied on open sharing of the study advert and was therefore self-selecting.

Eligible participants were aged 18 years or over and identified as Norwegian, Swedish, or British nationals. Participation was voluntary and anonymous.

A total of 399 participants took part. Responses from participants who did not complete the survey (*n* = 70) or requested data removal (*n* = 1) were excluded, resulting in a final sample of 328 participants: 98 from Norway, 115 from Sweden, and 115 from the United Kingdom. The target was approximately 100 participants per country, informed by practical constraints, prior research using comparable samples (e.g., Sarkissian et al., 2010; Shariff et al., 2014), and power considerations.

### 2.3. Procedure

The study was hosted on LimeSurvey. Participants first read an information sheet and provided informed consent before completing the survey. To reduce priming, the study’s aims were described in broad terms.

All study materials were available in English, Norwegian, and Swedish. Materials were translated from English into Norwegian and Swedish and then back-translated into English. Translation was completed by the first author, who is proficient in all three languages, in collaboration with one Norwegian teacher and one Swedish teacher.

Participants first provided demographic information, including age, gender, education, political orientation, religious belief, and previous contact with crime or the criminal justice system. They then read a vignette describing a fictional offence in which a healthcare professional murders an elderly man in his care following an argument over a card game. After reading the vignette, participants completed measures of sentence severity, retributive attitudes, and rehabilitation-related punishment. The Free Will Inventory was presented last to minimise the possibility that reflecting on free will beliefs would influence responses to the vignette.

### 2.4. Measures

#### 2.4.1. Demographic Questionnaire

Participants reported demographic information including age, gender, education, political orientation, religious belief, victimisation, and contact with the criminal justice system. These variables were collected because previous research suggests that factors such as political orientation, gender, and spirituality may relate to free will beliefs and punitive attitudes (e.g., Everett et al., 2021; Katz & Fondacaro, 2021).

#### 2.4.2. Vignette

Participants read a brief fictional vignette describing a homicide committed by a healthcare professional against an elderly man in his care.

#### 2.4.3. Retribution Scale

Support for retributive punishment was assessed using an adapted version of the Retribution Scale (Cullen et al., 1988), which has been used in previous research on punishment attitudes (e.g., Katz & Fondacaro, 2021; Koppel et al., 2018). Participants rated agreement with statements such as, “The more serious the offense is, the more a person deserves to be punished,” on a 7-point Likert scale from 1 (*strongly disagree*) to 7 (*strongly agree*). Katz and Fondacaro (2021) reported internal consistency of α = 0.83.

#### 2.4.4. Sentence Severity

Sentence severity was assessed using a single-item measure adapted from Katz and Fondacaro (2021): “How severely do you think the offender should be punished?” Responses were made on a 7-point Likert scale from 1 (*not at all severely*) to 7 (*very severely*).

#### 2.4.5. Retributive Punishment and Rehabilitation Measure

Participants were also asked to recommend further punishment after the offender had completed a two-year, 100% effective rehabilitation programme while incarcerated. This measure, adapted from Shariff et al. (2014), was intended to capture support for punishment beyond rehabilitation.

#### 2.4.6. Free Will Inventory

Free will beliefs were assessed using Part 1 of the Free Will Inventory (FWI; Nadelhoffer et al., 2014). This section comprises three 5-item subscales measuring belief in free will, determinism, and dualism. Only these three subscales were used in the present analyses. The FWI was placed at the end of the survey to reduce priming effects.

### 2.5. Data Analysis

Data were analysed to examine group differences and associations between free will beliefs and punishment attitudes. Analyses included ANOVA/MANOVA, ordinal regression, multiple regression, chi-square analysis, and multinomial logistic regression, depending on the hypothesis being tested.

### 2.6. Ethical Considerations

Ethical approval was granted by the University of Birmingham STEM Ethics Committee on 26 May 2022. The study was conducted in accordance with the British Psychological Society (2021).

Participants received information about the study before taking part and indicated consent electronically. They were informed that participation was voluntary, responses would be anonymised, and they could withdraw their data within one month of participation. All materials were provided in English, Norwegian, and Swedish to support accessibility and informed consent.

## 3. Results

### 3.1. Descriptive Statistics

Descriptive statistics for demographic variables and free will beliefs are presented in Table 1. The sample included 328 participants from the United Kingdom (*n* = 115), Norway (*n* = 98), and Sweden (*n* = 115). British participants were the only group with more women than men, whereas Norway and Sweden had more male than female participants. Across the full sample, most participants placed themselves between slightly left and slightly right politically, and most identified as not religious. Educational profiles differed across countries, with Norwegian participants most often reporting a master’s degree, British participants most often reporting a bachelor’s degree, and Swedish participants showing a broader spread across educational levels.

Mean Free Will Inventory scores also varied across countries. British participants had the highest mean scores for libertarian free will (M = 23.7), determinism (M = 14.0), and dualism (M = 22.2). Norwegian participants had the lowest mean libertarian free will score (M = 21.3), whereas Swedish participants had the lowest mean scores for determinism (M = 12.8) and dualism (M = 17.1).

### 3.2. Hypothesis 1

It was hypothesised that there would be significant differences between nationalities in beliefs in libertarian free will, determinism, and dualism, with Norwegian and Swedish participants expected to report lower belief in free will and dualism and higher belief in determinism than British participants. Preliminary assumption checks were conducted. Homogeneity of variance was met for libertarian free will and determinism, but not for dualism; accordingly, Welch’s ANOVA was used for the dualism analysis.

A MANOVA was conducted to examine the combined effect of nationality on the three FWI subscales. Pillai’s Trace indicated a significant multivariate effect of nationality, V = 0.09, approx. F = 5.38, *p* < 0.001. Follow-up univariate analyses showed significant differences by nationality for libertarian free will, F(2, 323) = 3.607, *p* = 0.02, and dualism, F(2, 323) = 13.86, *p* < 0.001, but not for determinism, F(2, 323) = 0.75, *p* = 0.47.

For libertarian free will, Tukey HSD post hoc tests indicated that British participants scored significantly higher than Norwegian participants (difference = 2.24, 95% CI [0.18, 4.30], *p* = 0.029). The differences between Swedish and Norwegian participants (difference = 1.82, 95% CI [−0.25, 3.88], *p* = 0.098) and between British and Swedish participants (difference = 0.42, 95% CI [−1.55, 2.40], *p* = 0.868) were not significant.

For determinism, a one-way ANOVA showed no significant differences between nationalities, F(2, 323) = 0.75, *p* = 0.47.

For dualism, Welch’s ANOVA showed a significant effect of nationality, F(2, 211.03) = 15.087, *p* < 0.001. Games–Howell post hoc tests showed that British participants scored significantly higher than both Norwegian participants (difference = 4.72, 95% CI [2.14, 7.30], *p* < 0.001) and Swedish participants (difference = 4.96, 95% CI [2.49, 7.44], *p* < 0.001). Norwegian and Swedish participants did not differ significantly (difference = −0.24, 95% CI [−2.83, 2.35], *p* = 0.97).

Overall, Hypothesis 1 was partly supported. British participants reported significantly higher dualism than both Norwegian and Swedish participants, and significantly higher libertarian free will than Norwegian participants, but no significant nationality differences were found for determinism.

### 3.3. Hypothesis 2

It was hypothesised that participants’ scores on the Free Will Inventory subscales would be predicted by nationality. Multiple regression analyses were conducted to examine whether nationality predicted scores on libertarian free will, determinism, and dualism after controlling for demographic and personal experience variables. These covariates were gender, political orientation, voting frequency, education level, religious belief, age, criminal record, personal experience of being a victim of crime, family experience of victimisation, and concern about future victimisation. Consistent with Hypothesis 1, nationality significantly predicted libertarian free will and dualism scores, but not determinism.

For libertarian free will, Swedish participants scored significantly higher than Norwegian participants (B = 2.407, SE = 0.89, t = 2.70, *p* = 0.007), and British participants also scored significantly higher than Norwegian participants (B = 3.514, SE = 0.93, t = 3.76, *p* < 0.001). The overall model was significant, F(28, 297) = 3.581, *p* = 1.945 × 10^−8^, explaining 25.24% of the variance (R^2^ = 0.2524, adjusted R^2^ = 0.1819).

For dualism, British participants scored significantly higher than Norwegian participants (B = 4.267, SE = 1.18, t = 3.616, *p* < 0.001), whereas Swedish participants did not differ significantly from Norwegian participants.

For determinism, nationality did not significantly predict scores. Neither the Swedish coefficient (B = −0.253, SE = 0.97, t = −0.260, *p* = 0.795) nor the British coefficient (B = 1.980, SE = 1.01, t = 1.942, *p* = 0.053) was statistically significant.

Overall, Hypothesis 2 was partly supported. Nationality significantly predicted libertarian free will and dualism, but not determinism. See Supplementary Materials Table S1.

### 3.4. Hypothesis 3

It was hypothesised that British participants would be more likely than Norwegian or Swedish participants to recommend harsher punishments. An ordinal regression analysis examined the relationship between nationality and sentence severity recommendations, measured on a 7-point scale from 1 (*not very severely*) to 7 (*very severely*). Nationality significantly predicted sentencing severity (B = 0.64, SE = 0.14, z = 4.55, *p* < 0.00001). The pattern indicated that Norwegian participants were least likely to recommend harsher punishments, followed by Swedish participants and then British participants. The corresponding odds ratio showed that British participants were 1.90 times more likely than Norwegian participants to recommend harsher punishments (OR = 1.905). Overall, Hypothesis 3 was supported.

### 3.5. Hypothesis 4

It was hypothesised that Norwegian and Swedish participants would place greater importance than British participants on understanding why a person commits a crime and on providing access to treatment, and that they would recommend less punitive sentences following rehabilitation. Ordinal regression analyses were conducted for the two rehabilitation-related attitude items. For understanding why a crime was committed, Norwegian participants placed significantly greater importance on this than British participants (B = 0.69, SE = 0.2598, z = 2.66, *p* = 0.007), whereas Swedish participants did not differ significantly from British participants (B = −0.37, SE = 0.23, z = −1.57, *p* = 0.11).

A similar pattern was found for access to treatment. Norwegian participants placed significantly greater importance on treatment access than British participants (B = 0.75, SE = 0.27, z = 2.80, *p* = 0.0051), whereas Swedish participants again did not differ significantly from British participants (B = −0.34, SE = 0.23, z = −1.43, *p* = 0.15).

A chi-square analysis was then conducted to examine whether post-rehabilitation sentencing recommendations varied by nationality. This association was not statistically significant, χ^2^ = 9.6, df = 6, *p* = 0.14, indicating that sentencing preferences following a 100% effective rehabilitation programme did not significantly differ across national groups. See Supplementary Materials Table S2.

Overall, Hypothesis 4 was partly supported. Norwegian participants placed greater importance than British participants on both understanding why a crime was committed and providing access to treatment, but no significant differences were found for Swedish participants or for post-rehabilitation sentencing recommendations.

### 3.6. Hypothesis 5

It was hypothesised that free will beliefs would predict sentencing severity recommendations. A multiple regression analysis examined whether the three FWI subscales predicted sentencing severity. The overall model was significant, F(3, 324) = 11.44, *p* < 0.001, with an adjusted R^2^ of 0.087. Higher libertarianism scores predicted more severe sentencing recommendations (B = 0.040, SE = 0.009, t = 4.44, *p* < 0.001), whereas higher determinism scores predicted less severe sentencing recommendations (B = −0.030, SE = 0.009, t = −3.37, *p* < 0.001). Dualism was not a significant predictor (B = 0.014, SE = 0.007, t = 1.90, *p* = 0.057).

Overall, Hypothesis 5 was partly supported. Stronger belief in libertarian free will was associated with harsher sentencing recommendations, whereas stronger belief in determinism was associated with less severe sentencing recommendations.

### 3.7. Hypothesis 6

It was hypothesised that free will beliefs would predict views on the importance of understanding why an offender committed a crime and the importance of providing access to treatment. A multiple regression analysis examined whether the three FWI subscales predicted the importance placed on understanding why a crime was committed. The overall model was significant, F(3, 324) = 4.659, *p* = 0.003, with an adjusted R^2^ of 0.032. Higher libertarianism scores predicted lower endorsement of understanding why the crime was committed (B = −0.040, SE = 0.014, t = −2.95, *p* = 0.003), whereas higher determinism scores predicted greater endorsement of this understanding (B = 0.027, SE = 0.013, t = 2.05, *p* = 0.041). Dualism was not a significant predictor (B = 0.018, SE = 0.011, t = 1.68, *p* = 0.093).

A second multiple regression examined the importance placed on access to treatment for offenders. This model was also significant, F(3, 324) = 8.671, *p* = 0.000015, with an adjusted R^2^ of 0.066. Higher libertarianism scores predicted lower endorsement of access to treatment (B = −0.060, SE = 0.012, t = −4.84, *p* < 0.001), whereas higher dualism scores predicted greater endorsement of treatment access (B = 0.024, SE = 0.010, t = 2.48, *p* = 0.014). Determinism was not a significant predictor (B = 0.008, SE = 0.012, t = 0.69, *p* = 0.48).

Overall, Hypothesis 6 was partly supported. Stronger libertarian free will beliefs were associated with lower endorsement of both understanding crime causation and access to treatment. Determinism was positively associated with understanding crime causation only, whereas dualism was positively associated with access to treatment only.

### 3.8. Hypothesis 7

It was hypothesised that free will beliefs would predict sentencing recommendations following a hypothetical scenario in which an offender had completed a 100% effective rehabilitation programme. A multinomial logistic regression analysis was conducted with post-rehabilitation sentencing recommendation as the outcome and “release immediately” as the reference category. Higher libertarianism scores significantly increased the likelihood of recommending release with a fine rather than immediate release (B = 0.170, SE = 0.046, t = 3.72, *p* < 0.001), half sentence rather than immediate release (B = 0.16, SE = 0.035, t = 4.64, *p* < 0.001), and maximum punishment rather than immediate release (B = 0.22, SE = 0.038, t = 5.91, *p* < 0.001). The strongest effect was observed for recommending maximum punishment. Determinism and dualism were not significant predictors in any comparison: for release with a fine, determinism B = 0.014, SE = 0.041, t = 0.33, *p* = 0.74 and dualism B = 0.004, SE = 0.036, t = 0.12, *p* = 0.90; for half sentence, determinism B = −0.035, SE = 0.032, t = −1.07, *p* = 0.28 and dualism B = 0.024, SE = 0.027, t = 0.87, *p* = 0.38; and for maximum punishment, determinism B = −0.021, SE = 0.035, t = −0.61, *p* = 0.54 and dualism B = 0.010, SE = 0.029, t = 0.33, *p* = 0.74. McFadden’s R^2^ was 0.21.

Overall, Hypothesis 7 was partly supported. Higher libertarian free will beliefs predicted more punitive post-rehabilitation sentencing recommendations, whereas determinism and dualism did not.

## 4. Discussion

This study examined the relationship between free will beliefs and attitudes towards punishment across participants from Norway, Sweden, and the United Kingdom. Overall, the findings provide tentative support for the view that national context is associated with both free will beliefs and punitive attitudes. In particular, stronger libertarian free will beliefs were consistently associated with harsher sentencing preferences, whereas determinism and dualism showed more mixed and context-dependent relationships. The cross-national findings also suggest some alignment between the philosophical orientation of justice systems and public attitudes, although this alignment was not straightforward across all three countries.

The first hypothesis predicted cross-national differences in Free Will Inventory (FWI; Nadelhoffer et al., 2014) scores. This was partly supported. British participants scored significantly higher than Norwegian participants on libertarian free will and significantly higher than both Norwegian and Swedish participants on dualism. No significant nationality differences were found for determinism. This broadly replicates previous work showing that free will beliefs vary across countries (Wisniewski et al., 2019). The British pattern is consistent with a justice context that places relatively strong emphasis on individual responsibility and punishment, whereas the Norwegian and Swedish systems have historically placed greater emphasis on rehabilitation and, particularly in Sweden’s case, more deterministic or consequentialist ideas about criminal behaviour (Juth & Lorentzon, 2010; R v. Kennedy, 2007).

One interesting aspect of the findings is that British participants were not significantly different from Swedish participants on libertarian free will, despite the substantial philosophical differences often drawn between the UK and Swedish systems. One possibility is that legal philosophy does not map neatly onto contemporary public attitudes. Public views are likely shaped not only by formal criminal justice principles but also by political discourse, media narratives, and current social concerns. In this respect, the Swedish findings may reflect the influence of recent concern about crime and safety rather than only the formal philosophical roots of the Swedish criminal justice system. This possibility is consistent with the idea that beliefs about agency and punishment are socially embedded rather than simply derived from abstract philosophical commitments.

The dualism findings may also help explain why British participants differed more clearly from the other groups on some dimensions than on others. Wisniewski et al. (2019) found that dualism may be more strongly related to free will beliefs than determinism. If some British participants are more likely to view the mind as distinct from physical and environmental constraints, this may support belief in agency even without rejecting all deterministic influences. In other words, dualism may provide a metaphysical basis for preserving responsibility in the face of causal explanations of behaviour. This interpretation fits the UK context, in which assumptions of agency and moral responsibility remain central to criminal law and sentencing (R v. Kennedy, 2007). However, the current findings cannot establish whether national legal philosophy shapes these beliefs, or whether both are shaped by broader cultural traditions.

The second hypothesis examined whether nationality predicted FWI subscale scores after controlling for other variables. This was again partly supported. Nationality predicted libertarian free will and dualism, but not determinism. This strengthens the interpretation that the cross-national effects were not reducible simply to demographic variation. At the same time, the models also indicated that nationality was only one influence among several. Political orientation, education, age, gender, and religiosity also appeared relevant to some aspects of free will belief. This is theoretically important because it suggests that beliefs about free will are not simply products of national culture or criminal justice context; rather, they likely emerge from the interaction of institutional, ideological, and personal influences.

The association between political orientation and libertarian free will beliefs is particularly noteworthy. More right-leaning participants scored higher on libertarian free will, which is broadly consistent with the emphasis that more conservative worldviews often place on personal responsibility, self-determination, and individual accountability. Similarly, the finding that dualism was associated with religiosity is unsurprising given the long-standing connection between religious and metaphysical beliefs about mind, soul, and agency. The finding that women scored higher on dualism may also fit broader evidence that women tend, on average, to report higher religiosity than men (Beit-Hallahmi & Argyle, 2014; Schnabel, 2018), although this interpretation should be treated cautiously. By contrast, determinism was not predicted by nationality and appears to have functioned differently from the other two subscales. This may reflect the fact that determinism, as measured by the FWI, does not map as directly onto attitudes about blame and punishment as libertarian free will does.

The third hypothesis predicted that British participants would recommend stricter punishment than Norwegian or Swedish participants. This was supported. British participants were more likely than Norwegian participants to recommend harsher punishment, with Swedish participants falling in between. This pattern is broadly consistent with the punitive and responsibility-focused orientation often associated with the UK system, as compared with the more rehabilitative and welfare-oriented approach in Norway. The result also fits the broader argument that support for punishment is linked not only to individual beliefs but also to social and institutional context.

However, the present data cannot determine whether Swedish participants’ relatively punitive responses reflected retributive attitudes, preventive concerns, or some combination of both. Support for longer or more severe sentences does not necessarily indicate that participants viewed punishment as deserved suffering; it may also reflect consequentialist concerns about incapacitation, public protection, or risk management. A person may therefore hold relatively deterministic beliefs about the causes of crime while still supporting restrictive sentences for serious or dangerous offenders. This distinction is important in the Swedish context, where recent work has described a punitive turn in criminal policy despite Sweden’s historically liberal and welfare-oriented penal tradition (Tham, 2026). The Swedish findings may therefore reflect not simply a disconnect between formal justice-system philosophy and public attitudes, but also a context in which public concern about crime and changing penal politics may be influencing one another.

That said, the Swedish result was less clear-cut than expected. Swedish participants were more punitive than Norwegian participants, despite the Swedish criminal justice tradition being more explicitly associated with deterministic and consequentialist thought (Juth & Lorentzon, 2010). This may again suggest that current public attitudes can diverge from the official or historical philosophy of a justice system. One possible explanation is that changing crime patterns and media salience shape punitive attitudes more immediately than abstract legal theory. Recent public concern about gang violence and firearm crime in Sweden may be relevant here (Hradilova Selin et al., 2024; SVT Nyheter, 2023). More generally, previous work suggests that exposure to wrongdoing or immoral behaviour can increase endorsement of free will and punitive attitudes (Clark et al., 2014). It is therefore plausible that perceptions of rising crime may increase support for harsher punishment even in contexts with less retributive formal traditions.

The fourth hypothesis concerned rehabilitation-related attitudes. This was only partly supported. Norwegian participants placed greater importance than British participants on understanding why a person committed a crime and on providing treatment, which aligns well with the rehabilitative emphasis of the Norwegian system (Stortinget, 2019). However, Swedish participants did not differ significantly from British participants on either measure. Again, this complicates any simple claim that public attitudes directly mirror the philosophical underpinnings of national justice systems. It may be that Norwegian penal culture is more clearly reflected in public attitudes than Swedish penal philosophy is, or that Swedish public attitudes are currently being shaped by countervailing social and political pressures.

The second part of Hypothesis 4 was not supported: post-rehabilitation sentencing recommendations did not differ significantly by nationality. This is an important result because it suggests that even where national groups differ in general punishment attitudes or views on treatment, they may still converge in believing that punishment can remain legitimate even after rehabilitation. One interpretation is that punishment serves symbolic or moral functions beyond prevention, such as affirming norms, expressing condemnation, or maintaining social cooperation (Walsh, 2006). Another possibility is that participants did not fully accept the premise of a “100% effective” rehabilitation programme, especially in the context of a serious violent offence. In that case, some may have continued to support punishment because they remained sceptical that complete rehabilitation was genuinely possible. Either way, the absence of a nationality effect here suggests that post-rehabilitation punishment may be shaped less by national context than by broader intuitions about desert.

The fifth hypothesis examined whether free will beliefs predicted sentencing severity. This was partly supported. Stronger libertarian free will beliefs predicted harsher sentencing, while stronger determinism predicted less severe sentencing. This is consistent with prior work linking free will belief to blame and punitive judgment (Appelbaum et al., 2015; Morse, 2000; Shariff et al., 2014). If individuals believe that offenders freely choose their actions, they may be more likely to view punishment as deserved. Conversely, if they place greater weight on causal determinants of behaviour, they may be less inclined to endorse severe punishment. The effect sizes were modest, however, and the model explained a relatively small proportion of variance, indicating that free will beliefs are only one part of a broader set of influences on punitive attitudes.

This limited explanatory power is not surprising. Punishment judgements are also shaped by emotional reactions to the offence, perceptions of dangerousness, offender characteristics, and beliefs about social order. Previous work has shown that high-affect crimes can reduce the difference in punitiveness between those who do and do not strongly endorse free will (Haynes et al., 2003; Krueger et al., 2014). The present study used a serious, high-affect vignette to maximise comparability across countries, yet free will beliefs still predicted sentencing severity. This suggests that beliefs about agency remained relevant even in the context of a crime likely to evoke strong emotional responses. At the same time, the modest variance explained reinforces the need not to overstate the role of metaphysical belief at the expense of other psychological and contextual factors. However, the specific features of the vignette may also have shaped participants’ responses. The offender was described as a healthcare worker, a role normally associated with care, trust, and protection, and the victim was an older adult. The violation of expected professional responsibility, together with the perceived vulnerability of the victim, may have intensified emotional reactions to the offence and increased support for punishment independently of participants’ free will beliefs.

The sixth hypothesis concerned the relationship between free will beliefs and support for understanding crime causation and providing treatment. This was also only partly supported. Stronger libertarian free will beliefs predicted lower endorsement of both understanding why the crime occurred and providing access to treatment. This is consistent with the broader literature suggesting that stronger free will beliefs are associated with greater emphasis on personal responsibility and less emphasis on situational or causal explanations (Appelbaum et al., 2015; Shariff et al., 2014). These findings strengthen the interpretation that libertarian free will beliefs are linked not only to harsher punishment, but also to lower support for rehabilitative or explanatory responses to crime.

Determinism showed a more selective pattern. It predicted greater endorsement of understanding why a crime was committed, but it did not predict support for treatment. This is an interesting divergence. One possible explanation is that participants with stronger deterministic beliefs may be more open to causal explanation without necessarily believing that offenders are amenable to change. In that sense, determinism may reduce blame without increasing confidence in rehabilitation. This interpretation fits with the idea that some deterministic worldviews may encourage fatalism as much as compassion. If participants see behaviour as strongly caused by stable biological, social, or developmental influences, they may view treatment as less likely to succeed. The findings therefore suggest that reducing endorsement of libertarian free will may not be the same thing as increasing confidence in rehabilitation.

Dualism again showed a more complicated pattern. It was associated with greater endorsement of treatment, but not with greater endorsement of understanding why the crime occurred. One possible explanation is that dualism does not simply track punitiveness or leniency, but reflects a distinct way of conceptualising the person. If some participants understand wrongdoing in terms of a mind or self that is partly separable from material causes, they may place less emphasis on analysing environmental determinants while still supporting interventions aimed at changing the person. This interpretation remains speculative, but it is broadly compatible with Wisniewski et al.’s (2019) finding that dualism is more closely tied to free will belief than determinism. More generally, the dualism findings highlight that the three FWI subscales do not operate as interchangeable proxies for one another.

The seventh hypothesis examined post-rehabilitation sentencing. This was partly supported. Stronger libertarian free will beliefs predicted greater endorsement of punitive sentencing even after a hypothetical 100% effective rehabilitation programme, whereas determinism and dualism did not. This finding is theoretically important because it suggests that libertarian free will belief is associated not merely with punishment as a means of prevention, but with punishment as something that may still be seen as justified even when prevention is no longer at issue. This is consistent with a retributive orientation, in which offenders are viewed as deserving punishment because of what they freely chose to do, or for the victim, rather than because punishment is needed to reduce future harm (Caruso, 2021; Morse, 2000). However, as noted earlier, punishment severity should not be treated as a direct proxy for punishment theory. Severe punishment may be justified on preventive or incapacitative grounds, while retributive punishment may be constrained by proportionality. The present findings therefore suggest an association between libertarian free will beliefs and more punitive sentencing preferences, but they cannot establish the precise justification participants had in mind when recommending punishment.

The absence of effects for determinism and dualism in this model may reflect conceptual limitations in how these constructs were measured. The libertarian free will subscale maps relatively directly onto responsibility and choice, whereas some determinism items may be interpreted in ways that are more cosmological or metaphysical than morally relevant. For example, endorsement or rejection of strong determinist statements may reflect beliefs about fate, science, or religion rather than clear views about criminal responsibility. This may help explain why determinism was less stable as a predictor across analyses. The findings therefore point not only to the importance of free will beliefs, but also to the need to distinguish carefully between different components of those beliefs when examining punishment attitudes.

Taken together, the clearest pattern in the findings concerns libertarian free will beliefs and punitive attitudes. Stronger libertarian free will beliefs were consistently associated with more punitive responses across sentencing severity, rehabilitation-related attitudes, and post-rehabilitation sentencing. Cross-nationally, British participants generally reported higher libertarian free will and dualism scores and greater punitiveness, particularly in comparison with Norwegian participants. By contrast, the distinctions between Norway and Sweden, and the roles of determinism and dualism, were more muted and less consistent than expected. This does not undermine the value of the cross-national comparison; rather, it suggests that the relationship between national justice context, metaphysical beliefs, and punishment attitudes is more complex than a simple alignment between legal philosophy and public opinion. The findings therefore indicate a relatively consistent individual-level association between libertarian free will belief and punitive attitudes, alongside a more tentative and context-dependent relationship between national setting and public beliefs about punishment.

### 4.1. Strengths and Limitations

A key strength of the study is its cross-national design. Much of the existing literature on free will beliefs and punishment has relied on single-country samples, making it difficult to examine whether such relationships generalise across different legal and cultural settings. By comparing participants from Norway, Sweden, and the United Kingdom, this study extends previous work and provides a more contextualised account of how beliefs about agency and punishment may vary across national settings. A further strength is the use of established measures, including the Free Will Inventory (Nadelhoffer et al., 2014), alongside multiple outcomes relating to both punishment severity and rehabilitation.

The study also considered a range of demographic and experience-related variables, which allowed the analyses to go beyond simple national comparisons. This made it possible to show that nationality remained relevant even after controlling for other factors, while also highlighting the importance of variables such as political orientation and religiosity. The inclusion of translated materials for Norwegian and Swedish participants was another strength, as it reduced the likelihood that the findings merely reflected English-language sampling.

Several limitations should also be acknowledged. First, the study relied on self-report data and vignette-based judgements, which may not reflect how participants would respond in real criminal justice settings (Field, 2009). Relatedly, the study relied on a single vignette involving a serious violent offence. Responses to murder may not reflect attitudes towards less serious or more common offences, as offence severity is likely to shape both punitive and rehabilitative attitudes. Even participants who generally support rehabilitation may endorse more severe punishment when the offence is especially serious. Future research should therefore examine whether the relationship between free will beliefs and punishment attitudes varies across offences of differing severity. Secondly, the design was cross-sectional, meaning that no causal conclusions can be drawn. The study cannot determine whether justice systems shape public beliefs, whether public beliefs shape justice systems, or whether both are influenced by broader political and cultural forces. Thirdly, although the materials were translated carefully and back-translated, the Norwegian and Swedish versions were not formally psychometrically validated. This limits confidence in strict cross-language equivalence.

A further limitation concerns the complexity of the constructs under study. Criminal justice systems are not philosophically uniform, and public attitudes do not necessarily track legal theory in any simple way. National comparisons therefore risk oversimplifying much more complex relationships between legal institutions, media environments, political discourse, and everyday beliefs. This is particularly relevant in interpreting the Swedish findings, which did not fit neatly with the expected pattern. Furthermore, several of the models explained a relatively modest proportion of variance, indicating that important predictors of punitive attitudes were not captured here. These may include emotional responses to crime, socioeconomic beliefs, media use, and more specific ideological commitments. Finally, although demographic and personal-experience variables were included as covariates in the analyses examining nationality as a predictor of FWI scores, the national groups were not perfectly matched. In particular, the British sample differed from the Norwegian and Swedish samples on some demographic and experience-related variables, including education and victimisation. These differences mean that cross-national comparisons should be interpreted cautiously, as some observed national differences may partly reflect sample composition rather than national context alone.

### 4.2. Implications and Future Research

The findings have practical implications because they suggest that punitive attitudes are related not only to beliefs about what works, but also to beliefs about what offenders deserve. If stronger libertarian free will beliefs are associated with harsher punishment and lower support for treatment, then public support for rehabilitative policy may depend partly on how criminal behaviour is framed. Greater public understanding of the social, developmental, and psychological causes of offending may encourage support for more rehabilitative and less purely retributive responses, although the present results also suggest that increasing determinist thinking alone may not be sufficient.

Future research could build on this study by examining how free will beliefs interact with emotional responses to crime, media exposure, political ideology, and religiosity. Experimental or longitudinal designs would be especially valuable for clarifying whether shifts in beliefs about agency lead to changes in punishment attitudes over time. It would also be useful to extend this work beyond Northern and Western Europe. Contexts with higher religiosity or more overtly punitive justice systems may reveal different relationships between free will beliefs, moral responsibility, and support for punishment (Berkessel et al., 2021). More broadly, future work should continue to distinguish between libertarianism, determinism, and dualism rather than treating belief in free will as a single construct.

## 5. Conclusions

This study found that stronger libertarian free will beliefs were consistently associated with harsher punitive attitudes, including in a scenario where rehabilitation was described as fully effective. Cross-national differences also emerged. British participants generally reported stronger free will-related beliefs and greater punitiveness than Norwegian participants, whereas Norwegian participants placed greater importance on understanding criminal behaviour and providing treatment. The Swedish findings were more mixed, suggesting that public attitudes do not map neatly onto the formal philosophy of a justice system. Overall, the clearest contribution of the study is the consistent association between libertarian free will beliefs and more punitive responses, alongside a more complex cross-national picture in which public attitudes appear to be shaped by both individual beliefs about agency and broader national context.

## Figures and Tables

**Table 1:** Demographic information.

Demographic	Britain (*n* = 115)	Norway (*n* = 98)	Sweden (*n* = 115)	Total
**Gender**				
Female	70 (60.9%)	41 (41.8%)	42 (36.5%)	153 (46.6%)
Male	43 (37.4%)	57 (58.2%)	72 (62.6%)	172 (52.4%)
Other	2 (1.7%)	0 (0.0%)	1 (0.9%)	3 (0.9%)
**Political Distribution**				
Very Left	15 (13.0%)	9 (9.2%)	12 (10.4%)	36 (11.0%)
Slightly Left	42 (36.5%)	38 (38.8%)	45 (39.1%)	125 (38.1%)
Centre	44 (38.3%)	30 (30.6%)	38 (33.0%)	112 (34.1%)
Slightly Right	14 (12.2%)	19 (19.4%)	17 (14.8%)	50 (15.2%)
Very Right	0 (0.0%)	2 (2.0%)	0 (0.0%)	2 (0.6%)
**Voting Frequency**				
Every election	51 (44.3%)	58 (59.2%)	72 (62.6%)	181 (55.2%)
Most elections	34 (29.6%)	23 (23.5%)	20 (17.4%)	77 (23.5%)
Half of elections	8 (7.0%)	9 (9.2%)	2 (1.7%)	19 (5.8%)
Less than half of elections	10 (8.7%)	1 (1.0%)	8 (7.0%)	19 (5.8%)
Never	12 (10.4%)	7 (7.1%)	13 (11.3%)	32 (9.8%)
**Education Level**				
Primary Education	0 (0.0%)	0 (0.0%)	6 (5.2%)	6 (1.8%)
Lower Secondary Education	3 (2.6%)	18 (18.4%)	20 (17.4%)	41 (12.5%)
Upper Secondary Education	40 (34.8%)	4 (4.1%)	30 (26.1%)	74 (22.6%)
Bachelor’s Degree	49 (42.6%)	27 (27.6%)	34 (29.6%)	110 (33.5%)
Master’s Degree	22 (19.1%)	45 (45.9%)	22 (19.1%)	89 (27.1%)
Doctoral Degree	1 (0.9%)	4 (4.1%)	3 (2.6%)	8 (2.4%)
**Religious Belief**				
Not Religious	88 (76.5%)	80 (81.6%)	88 (76.5%)	256 (78.0%)
Slightly Religious	20 (17.4%)	17 (17.3%)	24 (20.9%)	61 (18.6%)
Very Religious	7 (6.1%)	1 (1.0%)	3 (2.6%)	11 (3.4%)
**Criminal Record**				
Yes	3 (2.6%)	7 (7.1%)	4 (3.5%)	14 (4.3%)
No	112 (97.4%)	90 (91.8%)	108 (93.9%)	310 (94.5%)
I would rather not say	0 (0.0%)	1 (1.0%)	3 (2.6%)	4 (1.2%)
**Victim of a Crime**				
No	50 (43.5%)	52 (53.1%)	46 (40.0%)	148 (45.1%)
Yes	64 (55.7%)	43 (43.9%)	67 (58.3%)	174 (53.0%)
I would rather not say	1 (0.9%)	3 (3.1%)	2 (1.7%)	6 (1.8%)
**Free Will Beliefs**				
Libertarian Free Will (Avg.)	23.7 (67.7%)	21.3 (60.9%)	23.0 (65.7%)	22.66 (64.7%)
(Determinism (Avg.)	14.0 (40.0%)	13.4 (38.3%)	12.8 (36.6%)	13.4 (38.3%)
Dualism (Avg.)	22.2 (63.4%)	17.3 (49.4%)	17.1 (48.9%)	18.86 (53.9%)

## Data Availability

The data presented in this study are not publicly available due to ethical and privacy restrictions. Informed consent for data sharing was not obtained from participants, and the study’s ethical approval did not include permission for the dataset to be made available beyond the research team.

## Supplementary Materials

The following supporting information can be downloaded at: https://www.mdpi.com/article/10.3390/bs16060868/s1, Table S1: Nationality as a predictor for FWI part 1 scores; Table S2: Observed and Expected Frequencies of Punishment Recommendations by Country.
