# Supplementary material for: Exploring Free Will Beliefs and Attitudes Towards Punishment in Norway, Sweden and the United Kingdom
Source: Behav Sci (Basel). 2026 May 30;16(6):868. doi: 10.3390/bs16060868 (PMC13296269; doi:10.3390/bs16060868)
Supplement: Supplementary file 1 [file behavsci-16-00868-s001.zip › behavsci-4321827-supplementary.pdf]

Supplementary Material 1

Table S1. Nationality as a predictor for FWI part 1 scores

Predictor	Estimate (B)	SE	t	p
**Intercept**	12.59	4.14	3.04	0.003 **
Nationality (Swedish)	2.40	0.89	2.70	0.007 **
Nationality (British)	3.51	0.93	3.76	< .001 ***
Gender (Male)	-0.53	0.70	-0.76	0.44
Gender (Other)	-7.47	3.46	-2.16	0.032 *
Politics (Left)	1.22	1.15	1.06	0.29
Politics (Center)	3.75	1.17	3.18	0.002 **
Politics (Right)	6.73	1.36	4.94	< .001 ***
Politics (Very Right)	9.86	2.95	3.33	0.001 ***
Votefreq (Most Elections)	-1.21	0.84	-1.42	0.15
Votefreq (Half Elections)	-1.84	1.49	-1.23	0.21
Votefreq (Less Half)	-1.76	1.47	-1.20	0.23
Votefreq (Never)	-1.12	1.18	-0.94	0.34
Education (Lower Secondary)	5.63	2.63	2.14	0.033 *
Education (Upper Secondary)	3.34	2.57	1.30	0.19
Education (Bachelor's)	2.70	2.53	1.06	0.28
Education (Master's)	1.91	2.58	0.74	0.45
Education (Doctoral)	4.32	3.27	1.32	0.18
Religious (Slightly)	2.47	1.97	1.25	0.21
Religious (Not)	2.88	1.84	1.56	0.11
Age	-0.0003	0.02	-0.009	0.99
Criminalrec (No)	-0.83	1.71	-0.48	0.62
**Predictor**	**Estimate (B)**	**SE**	**t**	**p**

Predictor	Estimate (B)	SE	t	p
Criminalrec (Rather NS)	-6.80	3.49	-1.94	0.053
Victcrime (No)	0.27	0.73	0.36	0.71
Victcrime (Rather NS)	1.60	2.79	0.57	0.56
Famvictcrime (No)	1.73	0.73	2.34	0.019 *
Famvictcrime (Rather NS)	-3.98	1.91	-2.08	0.038 *
Worriedfuturevictim (Some)	0.60	1.32	0.45	0.64
Worriedfuturevictim (No)	0.57	1.37	0.41	0.67

Residual standard error: 5.79 on 297 degrees of freedom

Multiple R-squared: 0.2524

Adjusted R-squared: 0.1819

F-statistic: 3.581 on 28 and 297 DF, p-value: 1.945e-08

Supplementary Material 2

Table S2. Observed and Expected Frequencies of Punishment Recommendations by Country

Country	Release Immediately	Release with Fine	Half Sentence	Maximum Punishment	Total
**Norwegian**	11 (9.93)	9 (7.83)	50 (42.74)	28 (29.50)	98
**Swedish**	7 (11.37)	9 (8.96)	46 (48.92)	53 (33.76)	115
**British**	15 (11.70)	8 (9.22)	46 (50.34)	46 (34.74)	115
**Total**	33	26	142	127	328

Model Fit Indicators

Statistic	Value
Chi-squared (χ^2)	9.6
Degrees of Freedom	6
p-value	0.14
